# SNP Association Mapping across the Extended Major Histocompatibility Complex and Risk of B-Cell Precursor Acute Lymphoblastic Leukemia in Children

**DOI:** 10.1371/journal.pone.0072557

**Published:** 2013-08-22

**Authors:** Kevin Y. Urayama, Anand P. Chokkalingam, Catherine Metayer, Helen Hansen, Suzanne May, Patricia Ramsay, Joseph L. Wiemels, John K. Wiencke, Elizabeth Trachtenberg, Pamela Thompson, Yasushi Ishida, Paul Brennan, Kent W. Jolly, Amanda M. Termuhlen, Malcolm Taylor, Lisa F. Barcellos, Patricia A. Buffler

**Affiliations:** 1 School of Public Health, University of California, Berkeley, Berkeley, California, United States of America; 2 Center for Clinical Epidemiology, St. Luke’s Life Science Institute, Tokyo, Japan; 3 Laboratory for Molecular and Neuroepidemiology, University of California San Francisco, San Francisco, California, United States of America; 4 Genetic Epidemiology and Genomics Laboratory, University of California, Berkeley, Berkeley, California, United States of America; 5 Center for Genetics, Children’s Hospital Oakland Research Institute, Oakland, California, United States of America; 6 Cancer Immunogenetics, University of Manchester, St. Mary’s Hospital, Manchester, United Kingdom; 7 Department of Pediatrics, Ehime Prefectural Center Hospital, Ehime, Japan; 8 Genetic Epidemiology Group, International Agency for Research on Cancer, Lyon, France; 9 Department of Pediatrics, Kaiser Permanente, Roseville, California, United States of America; 10 Department of Pediatrics, Keck School of Medicine, University of Southern California, Los Angeles, California, United States of America; 11 Jonathan Jaques Children’s Cancer Center, Miller Children’s Hospital, Long Beach, California, United States of America; 12 Handforth, Cheshire, United Kingdom; University of Thessaly, Faculty of Medicine, Greece

## Abstract

The extended major histocompatibility complex (xMHC) is the most gene-dense region of the genome and harbors a disproportionately large number of genes involved in immune function. The postulated role of infection in the causation of childhood B-cell precursor acute lymphoblastic leukemia (BCP-ALL) suggests that the xMHC may make an important contribution to the risk of this disease. We conducted association mapping across an approximately 4 megabase region of the xMHC using a validated panel of single nucleotide polymorphisms (SNPs) in childhood BCP-ALL cases (n=567) enrolled in the Northern California Childhood Leukemia Study (NCCLS) compared with population controls (n=892). Logistic regression analyses of 1,145 SNPs, adjusted for age, sex, and Hispanic ethnicity indicated potential associations between several SNPs and childhood BCP-ALL. After accounting for multiple comparisons, one of these included a statistically significant increased risk associated with rs9296068 (OR=1.40, 95% CI=1.19-1.66, corrected p=0.036), located in proximity to *HLA-DOA*. Sliding window haplotype analysis identified an additional locus located in the extended class I region in proximity to *TRIM27* tagged by a haplotype comprising rs1237485, rs3118361, and rs2032502 (corrected global p=0.046). Our findings suggest that susceptibility to childhood BCP-ALL is influenced by genetic variation within the xMHC and indicate at least two important regions for future evaluation.

## Introduction

Acute lymphoblastic leukemia (ALL) is a clonal disorder involving the dysregulated proliferation of genetically altered lymphoid progenitor cells that lack the ability for differentiation and maturation. In children, B-cell precursor (BCP) ALL is the most common ALL subtype and accounts for about 80% of childhood ALL cases in most economically developed countries. BCP-ALL, which demonstrates a unique age-incidence peak between 2 and 5 years of age, is widely suspected to be caused by environmental exposures, though these have yet to be definitively identified [1]. Foremost among these are thought to be factors such as the effect of timing of exposure to infectious agents leading to inappropriate immune responses, in conjunction with variants in genes of the immunological pathway and early lymphoid development [2].

The extended major histocompatibility complex (xMHC) region, spanning about 7.6 megabases (Mb) on the short arm of chromosome 6 (6p21.3), is densely populated with genes that are critical to both innate and adaptive immunity in humans [3]. The xMHC is divided into five sub-regions consisting of the extended class I region at the telomeric end, and successively the classical class I, III, and II clusters bounded by an extended class II region at the centromeric end. Historically, genetic association studies of the xMHC have focused on the classical human leukocyte antigen (HLA) genes of the class I (*HLA-A*, *B*, and *C*) and class II (*HLA-DP*, *DQ*, and *DR*) regions. These encode cell surface glycoproteins that selectively bind and present, in allele selective fashion, processed antigenic peptides to T lymphocytes that initiate T-cell responses. While *HLA* genes are among the most polymorphic in humans, they account for only a small proportion of over 250 expressed xMHC loci, which include genes encoding cytokines, complement factors, and various others involved in critical cellular processes.

Evidence of susceptibility associated with xMHC loci has been identified in several autoimmune, malignant and infectious diseases, including asthma, Hodgkin and non-Hodgkin lymphoma, hepatitis B and HIV infection and others [4,5,6,7]. Despite evidence of linkage between predisposition to retrovirus-induced leukemia and the murine MHC, attempts to identify associations between childhood ALL and classical HLA alleles have been inconsistent largely due to study design limitations [8,9,10,11,12]. Although these have been largely overcome by the application of high-resolution HLA molecular genotyping to carefully ascertained case-control series, strong and replicable associations have yet to emerge.

A previous analysis of MHC SNP data and imputed HLA class I and II alleles derived from a childhood ALL genome-wide association study (GWAS) suggested that MHC genetic variation is unlikely to be a major determinant of BCP-ALL [13]. Nonetheless, a modest SNP association noted in the HLA class II region, together with strong positive findings from the largest study to date utilizing directly typed HLA genotypes [9], suggested that further examination of the xMHC in childhood ALL was warranted in a well-defined case-control series. We report here the results of SNP association mapping across a 4 Mb stretch of the xMHC spanning all major class I, II, and III loci using a validated SNP panel in a large sample of non-Hispanic white and Hispanic BCP-ALL cases (n=567) and controls (n=892) enrolled in the Northern California Childhood Leukemia Study (NCCLS).

## Materials and Methods

### Ethics Statement

The study protocol was approved by the Institutional Review Boards of the University of California, Berkeley and all collaborating institutions (California Department of Public Health, University of California, Davis, University of California, San Francisco, Children’s Hospital of Central California, Lucile Packard Children’s Hospital, Children’s Hospital and Research Center, Oakland, Kaiser Permanente, Roseville, Kaiser Permanente, Santa Clara, Kaiser Permanente, San Francisco, Kaiser Permanente, Oakland), and written informed consent was obtained from the parents or guardians on behalf of the children participants involved in this study. This study was conducted in accordance with the Declaration of Helsinki.

### Study participants

The current study was conducted within the NCCLS, an ongoing case-control study of childhood leukemia. Beginning in 1995, newly diagnosed childhood leukemia cases were ascertained at the time of diagnosis from major pediatric hospitals in a 17-county San Francisco Bay Area study region, expanded in 1999 to 35 counties in Northern and Central California, USA. Comparison with the California Cancer Registry (1997-2003) showed that the NCCLS case ascertainment protocol has captured about 95% of children diagnosed with leukemia in the participating study hospitals. For each eligible case, statewide birth records maintained by the California Office of Vital Records were utilized to generate a list of randomly selected controls that matched the case on child’s date of birth, sex, Hispanic ethnicity (a biological parent who is Hispanic), and maternal race. Information obtained through the birth certificates and commercially available searching tools were used to trace and enroll one or two matched controls for each case.

Cases and controls were considered eligible if they were under 15 years of age at date of diagnosis for cases (or corresponding reference date for controls), residents of the study region, had a biological parent who spoke either English or Spanish, and had no prior history of malignancy. Approximately 85% of eligible cases and 86% of eligible controls consented to participate [14]. A detailed description of control selection in the NCCLS is reported elsewhere [14,15].

In the current study, non-Hispanic white and Hispanic children with ALL and control children, recruited between 1995 and 2008 (study phases 1-3), were included in the analysis. These are the two largest racial/ethnic groups which together comprise about 85% of enrolled subjects. Other ethnic groups were excluded due to the small number of subjects. Children were classified as Hispanic if at least one biological parent self-identified as Hispanic. Children were assigned to the non-Hispanic white group if both biological parents self-identified as non-Hispanic white. In a previous NCCLS analysis, genetic admixture was assessed using a series of 80 ancestry informative markers for a subset of the cases and controls [16], and estimates of genetic ancestry (percent of European, Amerindian, and African ancestry) were determined. Comparison of these estimates between cases and matched controls showed no significant differences [16]. For the current study, a total of six hundred and eighty-eight ALL cases and 1,012 controls were considered, of which 635 cases (92.3%) and 915 controls (90.4%) had a DNA sample available for genotyping.

### DNA samples

Buccal cells as a source of DNA were obtained from case and control children using cytobrushes by trained interviewers. Cytobrushes were processed within 48 hours of collection by heating in the presence of 0.5N NaOH. Isolated DNA was later re-purified either manually using Gentra Puregene reagents (QIAGEN, USA, Valencia, CA) or an automated organic DNA extraction protocol (AutoGen, Holliston, MA). Whole genome amplification (WGA) was performed using GenomePlex reagents (Rubicon Genomics, Ann Arbor, MI) according to the manufacturer’s protocol. WGA products were cleaned with a Montage PCR9 filter plate (Millipore, Billerica, MA). When buccal cytobrush DNA was inadequate or not available (26.6% of subjects), DNA was isolated from dried bloodspots collected at birth and archived at ˗20ºC by the Genetic Disease Screening Program of the California Department of Public Health. After extraction using the QIAamp DNA Mini Kit (QIAGEN, USA, Valencia, CA), DNA samples were whole-genome amplified using REPLI-g reagents (QIAGEN, USA, Valencia, CA). Regardless of source, DNA specimens were quantified using human-specific Alu-PCR to confirm a minimum level of amplifiable human DNA [17].

### MHC genotyping

Genotyping was conducted using the Illumina MHC Mapping Panel (Illumina Inc., San Diego, CA) which comprises 1,293 SNPs spanning an approximately 4 Mb region of the xMHC bounded by the tripartite motif containing protein 27 (*TRIM27*) and motilin (*MLN*) genes at the telomeric and centromeric ends, respectively (NCBI Build 36). There is an average 3.8 kilobase (kb) spacing between each SNP, covering all major regions of the xMHC, including the classical class I, II and III regions, the extended class II region and part of the extended class I region. The panel set was designed with a strong emphasis on haplotype tagging SNPs which are highly informative of SNPs in strong linkage disequilibrium (LD). The chance of detecting an association is significantly influenced by the ability of a SNP or combination of SNPs to adequately represent the haplotypic diversity of the region. Genotyping was performed utilizing the robust Golden-Gate technology in a 96-well format on a 1,536 Sentrix Array Matrix [18]. It was shown previously in NCCLS subjects that when analyzed using Golden-Gate genotyping, buccal cell WGA DNA yielded genotypes that are highly concordant with those from genomic DNA from peripheral blood [19].

Genotyping was conducted on 1,550 unique DNA samples (635 cases and 915 controls), in addition to 10 sets of *Centre d’Etude du Polymorphisme Humain* (CEPH) family trios and duplicates of 10% of study samples. For quality control purposes, 113 SNPs that successfully genotyped in less than 90% of samples were excluded, as well as 30 SNPs that deviated from Hardy Weinberg equilibrium (p<0.01) in both non-Hispanic white and Hispanic controls, and 5 additional SNPs with a minor allele frequency of less than 0.01 in both non-Hispanic white and Hispanic controls.

Quality control metrics applied to the 1,550 samples also resulted in the exclusion of 17 samples (1.1%) with less than 95% overall genotyping success rate and 20 samples (1.3%) that showed questionable concordance between reported gender and gender prediction by the Illumina platform. There was 99.6% concordance of successfully genotyped SNPs in the duplicate series and a 0.2% Mendelian error rate was observed in the CEPH family trios. Application of these quality control criteria and a focus on BCP-ALL (54 T-cell or mixed lineage ALL cases excluded) resulted in the analysis of 1,145 SNPs in a total of 567 BCP-ALL cases and 892 controls (Table 1). Data are available on request in accordance with the policies and procedures of the NCCLS.

**Table 1:** Characteristics of childhood BCP-ALL cases and controls.

	Cases		Controls	
	n	%	n	%
**Total**	567	100	892	100
**Sex**				
Male	298	52.6	495	55.5
Female	269	47.4	397	44.5
**Age (years)**				
0-1	51	9.0	111	12.4
2-5	340	60.0	460	51.6
6-10	114	20.1	210	23.5
11-14	62	10.9	111	12.4
**Self-reported race/ethnicity**				
White, non-Hispanic	241	42.5	426	47.8
Hispanic	326	57.5	466	52.2
**BCP-ALL subtype** ^a^				
cALL^b^	309	49.8	NA	NA
Non-cALL	258	41.5	NA	NA
High-hyperdiploid	178	28.7	NA	NA
*TEL-AML1*	96	15.5	NA	NA
Normal karyotype	58	9.3	NA	NA

Abbreviations: BCP-ALL, B-cell precursor acute lymphoblastic leukemia; cALL, common ALL; NA, not applicable

^a^ Categories of ALL subtypes are not mutually exclusive. Percentages do not equal to 100. Cytogenetic data were available for 87% of patients.

^b^ cALL is defined as CD10+ and CD19+ ALL diagnosed between age 2-5 years.

For a large subset of the BCP-ALL cases (87%), data on hyperdiploidy and *TEL-AML1* chromosomal translocation were available as described in detail previously [20]. Subtypes of the cases included 309 common ALL (cALL, defined as CD10+ and CD19+ ALL aged 2 to 5 years), 178 high-hyperdiploid (51-67 chromosomes), 96 positive for the *TEL-AML1* chromosomal translocation, and 58 BCP-ALL with normal karyotypes. These non-mutually exclusive subtype groupings were used to examine potential subtype-specific effects for the final set of associated SNPs.

### Statistical analysis

Data analysis included a two-stage approach. First, we examined the contribution of 1,145 xMHC SNPs individually. We used logistic regression to calculate the odds ratio (OR) and 95% confidence intervals (CI) for each SNP adjusting for child’s age, sex, and race/ethnicity (i.e. non-Hispanic white or Hispanic). Various genetic models of inheritance were considered including log-additive, dominant, and recessive models, in addition to an evaluation of the dominance deviation from additivity. SNPs showing a nominal p-value of less than 0.01 in any of these analyses were considered potentially associated with childhood ALL and were subject to further analysis. Multivariable logistic regression was used to evaluate the independence of effect of multiple potentially associated SNPs within a region on childhood ALL risk. Stratified analyses by age (0-5 and 6-14 years) and gender were considered in sub-analyses of the data. To account for multiple comparisons in the presence of LD between SNPs, we calculated adjusted p-values based on 10,000 permutations of case-control status on 1,145 SNPs and considered adjusted p-values below a family-wise type I error rate threshold of 0.05 to be statistically significant.

**Figure 1:**
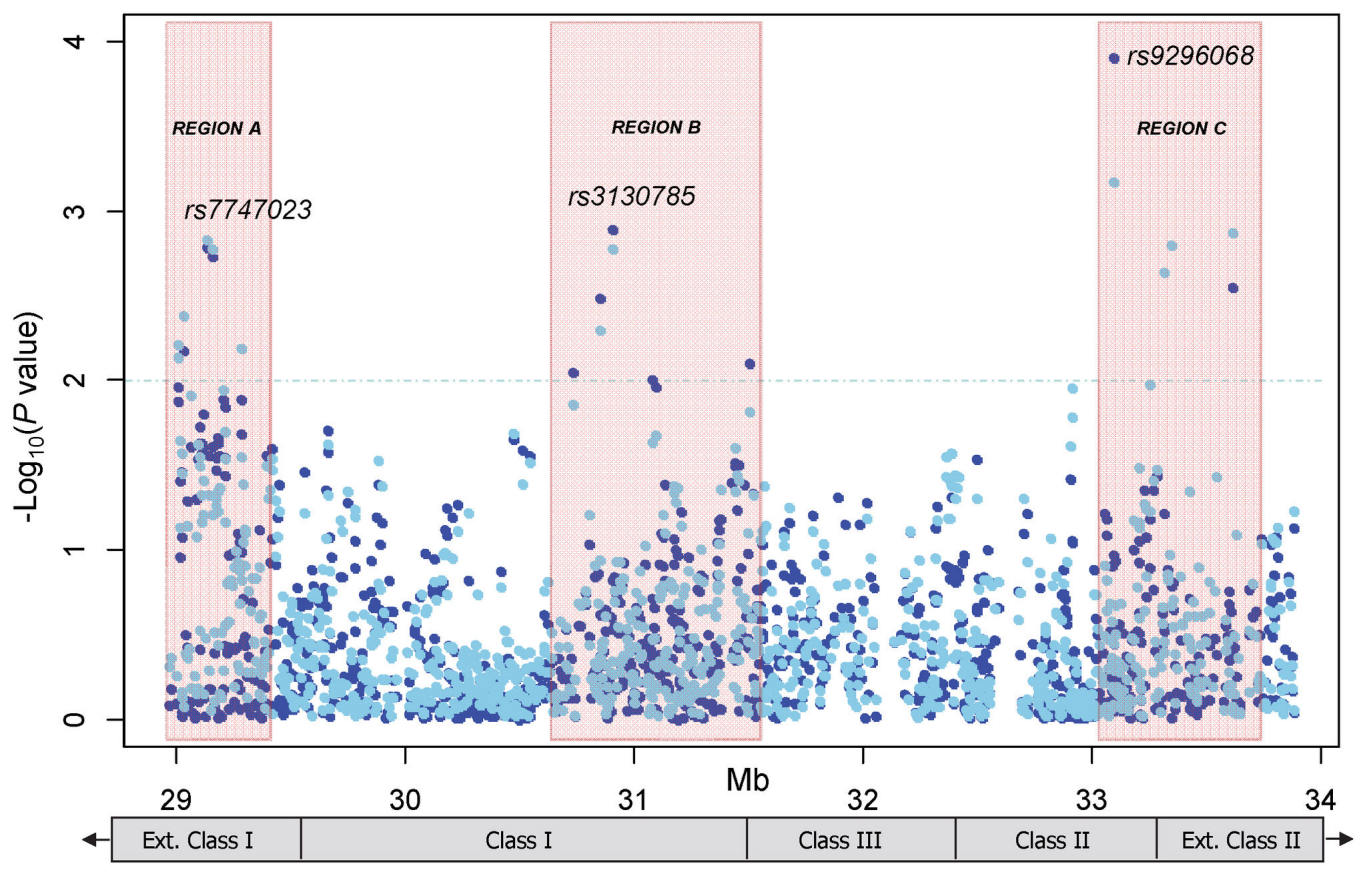
Analysis of 1,145 SNPs across a 4 Mb region of the extended major histocompatibility complex and risk of childhood BCP-ALL. Presented are -log_10_(p-values) resulting from the logistic regression analysis assuming log-additive (navy blue) and dominant (light blue) genetic models of inheritance and adjusting for child’s age, sex, and race/ethnicity. Results plotted above the dotted line represent nominal p-values of less than 0.01. Analyses evaluating the recessive and genotypic genetic models were also performed (not plotted) resulting in five additional SNPs with a nominal p-value of less than 0.01 which were also located within one of the three designated regions (Regions A-C). A total of 20 SNPs with a p-value below this threshold were considered in further analyses.

Second, we conducted three-SNP sliding window haplotype analyses across candidate regions selected by the location of SNPs that showed nominal p-values of less than 0.01. This resulted in 3 broad regions (Figure 1) for the haplotype analysis: 1) a region bound by rs381808 and rs3117330 referred to as region A (~364 kb region, extended class I); 2) a region bound by rs1264419 and rs3828886 referred to as region B (~864 kb region, class I-class III); and 3) a region bound by rs516535 and rs210134 referred to as region C (~598 kb region, class II-extended class II). In total, the haplotype sliding window analysis was performed on 395 genotyped SNPs (393 3-SNP haplotypes) located within these 3 regions. Haplotypes were predicted and reconstructed for each individual and frequencies estimated using the expectation-maximization algorithm based on unphased genotype data. Haplotypes with a frequency of less than 0.01 were grouped into one category. For each three-SNP sliding window, a global likelihood ratio test of association was performed to test the null hypothesis of no effect of any haplotype at that position. The permutation method was used to adjust for multiple comparisons for the 393 haplotype windows tested. Haplotype-specific effects were evaluated by modeling individual haplotype probabilities in a logistic regression assuming a log-additive effect of the haplotype and adjusting for age, sex, and race/ethnicity. Analyses were conducted using PLINK, UNPHASED version 3.1.4, and Haploview [21,22,23].

## Results

Case and control distributions for sex, age, and race/ethnicity were similar, as expected from the matched design of the NCCLS (Table 1). Hispanics comprised about 58% of cases and 52% of controls.

Analysis of 1,145 SNPs in childhood BCP-ALL, assuming a log-additive genetic model of inheritance, showed a quantile-quantile (Q-Q) plot of the expected versus observed –log_10_ p-value distribution that suggested little evidence of inflation in results caused by systematic error (Figure S1). Twenty SNPs were associated with a nominal p-value of less than 0.01 (log-additive, genotypic, dominant, and/or recessive genetic models), many of which were in LD (Figure 1 and Figure S2). These SNPs (Figure 1) appeared to cluster within three specific regions (designated A, B, and C) which served as the focus of the haplotype analysis. SNPs showing the strongest evidence of association based on p-value within each of the three regions included rs7747023 (OR=0.73, 95% CI=0.60-0.89, p=1.7x10^-3^) of region A, rs3130785 (OR=1.45, 95% CI=1.16-1.82, p=1.3x10^-3^) of region B, and rs9296068 (OR=1.37, 95% CI=1.17-1.61, p=1.2x10^-4^) of region C. Multivariable analyses evaluating the independence of associations between the 20 SNPs on BCP-ALL risk (Table S1) resulted in 6 SNPs that maintained low p-values and minimally attenuated risk estimates (Table 2). Stratified analysis showed no remarkable gender- or age-specific associations (Figure S3). The final multivariable model including all 6 SNPs and correcting for multiple comparisons showed a statistically significant association between rs9296068 and BCP-ALL risk (OR=1.40, 95% CI=1.19-1.66, corrected p=0.036).

**Table 2:** Association between xMHC genetic variants and BCP-ALL in children.

				Frequency^a^		Single SNP			Mutually adjusted			
SNP	Position	Region of xMHC	Minor allele	Cases	Controls	OR	95% CI^b^	p-value	OR	95% CI^b^	p-value	p-value _*(FWE)*_^c^
rs7747023	29133659	Extended class I	G	0.17	0.21	0.73	(0.60-0.89)	1.7x10^-3^	0.72	(0.59-0.88)	1.4x10^-3^	0.518
rs3130785	30904717	Class I	A	0.14	0.11	1.45	(1.16-1.82)	1.3x10^-3^	1.37	(1.09-1.74)	7.9x10^-3^	0.973
rs1632856	31079715	Class I	A	0.25	0.29	0.80	(0.68-0.95)	9.9x10^-3^	0.79	(0.66-0.94)	8.6x10^-3^	0.898
rs2524279	31500885	Class I	G	0.11	0.15	0.73	(0.58-0.92)	7.9 x10^-3^	0.70	(0.55-0.89)	3.0x10^-3^	0.749
rs9296068	33096673	Class II	C	0.42	0.36	1.37	(1.17-1.61)	1.2x10^-4^	1.40	(1.19-1.66)	5.7x10^-5^	0.036
rs213203^d^	33346382	Extended class II	A	0.47	0.49	0.68	(0.55-0.84)	3.6x10^-4^	0.69	(0.55-0.86)	7.4x10^-4^	0.347

Abbreviations: CI, confidence interval; FWE, family-wise type I error; OR, odds ratio; SNP, single nucleotide polymorphism; xMHC, extended major histocompatibility complex

^a^ Frequency of the minor allele in case and control subjects

^b^ ORs and 95% CI for each SNP in the single SNP analysis were derived using logistic regression assuming a log-additive genetic model of inheritance and adjusting for child’s age, sex, and race/ethnicity (non-Hispanic white versus Hispanic). The mutually adjusted analysis included additional adjustment for the effects of all other SNPs in the table.

^c^ Adjustment for multiple testing was performed with 10,000 permutations of case-control status on 1,145 SNPs and using FWE rate of 0.05. An adjusted p-value of less than 0.05 was considered statistically significant.

^d^ Evaluation of the genetic model of inheritance indicated a significant deviation from the log-additive model with an effect associated with heterozygotes. ORs and 95% CI were estimated for heterozygous genotypes compared to homozygous genotypes.

**Figure 2:**
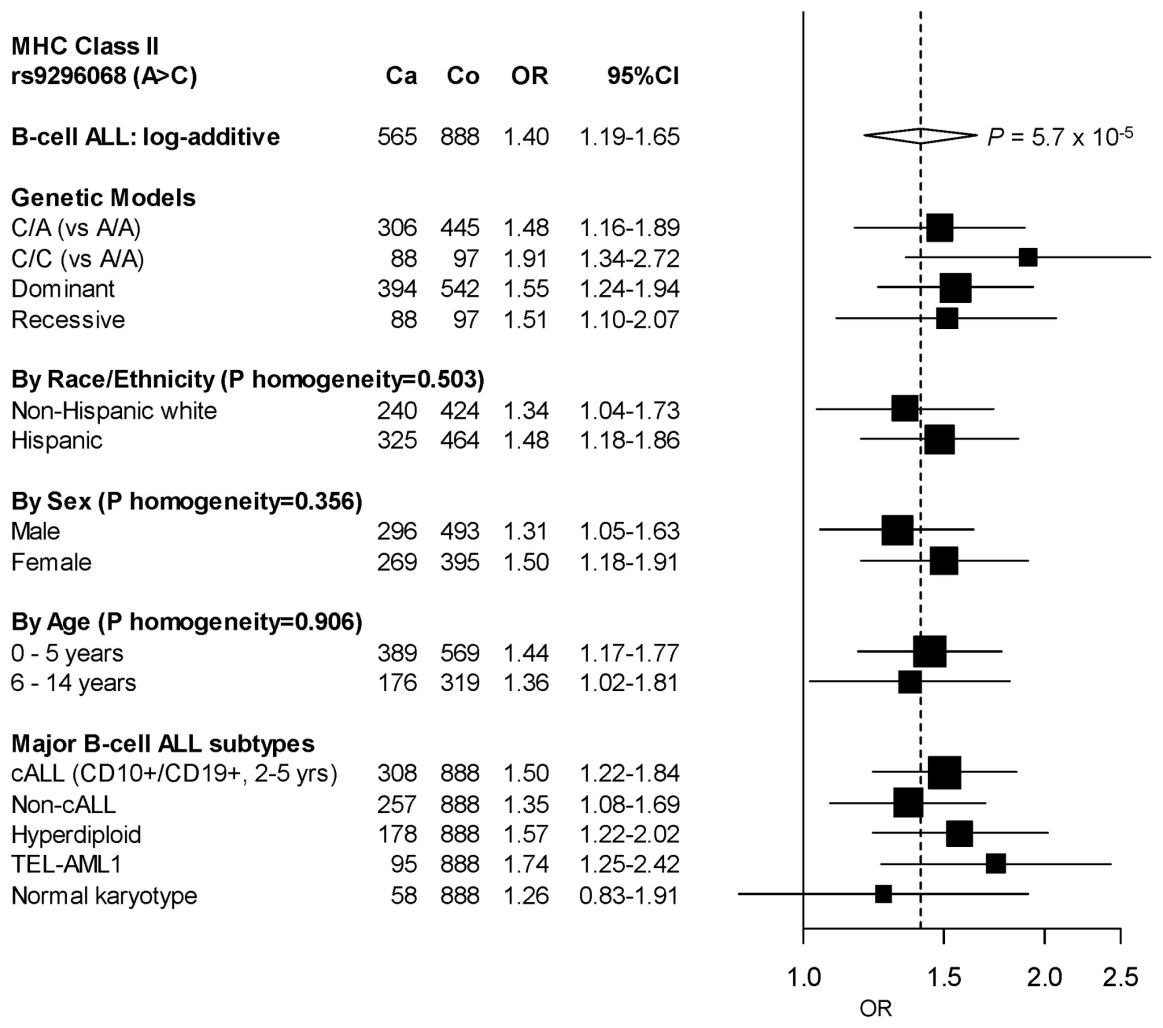
Stratified analysis of childhood BCP-ALL and the SNP rs9296068 by race/ethnicity, sex, and age group, and subgroup analyses by major subtypes. Odds ratios (ORs, represented by boxes with the area of each box inversely proportional to the variance of the estimate) and 95% CI (error bars) were derived using logistic regression assuming a log-additive genetic model and adjusting for rs7747023, rs3130785, rs1632856, rs2524279, and rs213203 (other potentially associated SNPs presented in Table 1) and additionally for child’s age, sex, and race/ethnicity based on the stratification variable. The dashed vertical line represents the OR of the SNP in the analysis of BCP-ALL among all subjects and the width of the diamond is the corresponding 95% CI. *P*_homogeneity_ was on the basis of the Cochran’s Q test statistic. Abbreviations: Ca, number of case; cALL, common acute lymphoblastic leukemia; Co, number of controls.

Further examination of SNP rs9296068 (Figure 2) showed little evidence of heterogeneity in effect between non-Hispanic white and Hispanic children (p=0.503), males and females (p=0.356), and children aged zero to five and aged six to fourteen (p=0.906). The risk estimates appeared consistently elevated for the two main cytogenetic subtypes (i.e. *TEL-AML1*-positive and high hypderdiploidy), but not for normal karyotype BCP-ALL (Figure 2, OR=1.26, 95% CI=0.83-1.91).

**Figure 3:**
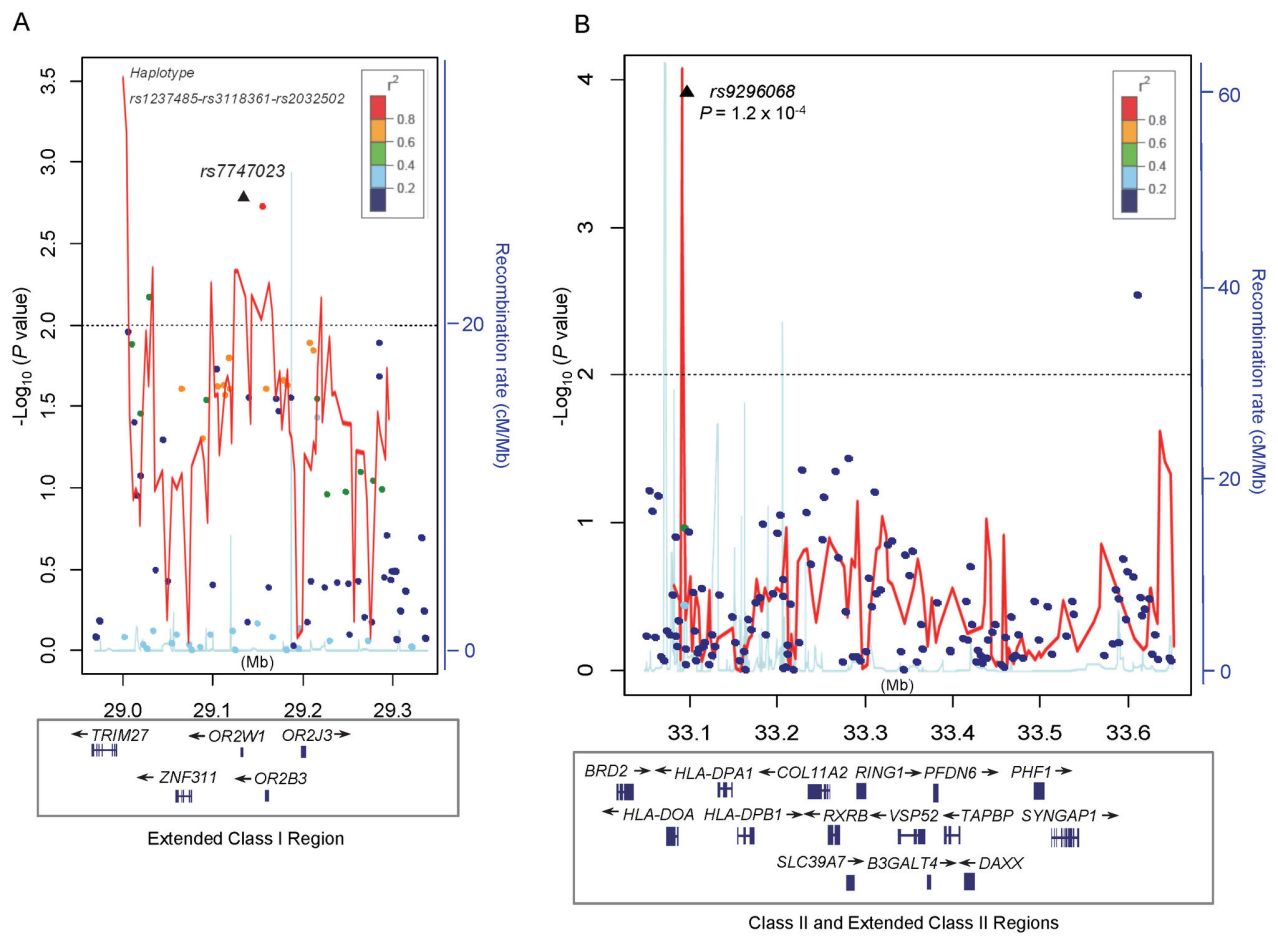
Plots of the two associated loci showing the results for the analysis of childhood BCP-ALL and individual SNPs (points) and three-SNP sliding window haplotypes (red lines). The -log_10_ (p-values) for each SNP (y-axis) are plotted against their chromosomal position (x-axis, Mb). The colors of the points indicate the degree of linkage disequilibrium (based on *r* ^*2*^) in relation to the index SNP (indicated by a black triangle). Results of the global likelihood ratio test of each three-SNP sliding window haplotype analysis are plotted and connected by the red lines. The plotted lines in blue are recombination rates (cM/Mb) based on HapMap Phase I and II data (http://hapmap.ncbi.nlm.nih.gov). A) Region A is indexed by rs7747023 as the strongest associated SNP. A statistically significant haplotype comprising rs1237485, rs3118361, and rs2032502 located adjacent to *TRIM27* was found to be associated with childhood BCP-ALL. B) Region C is indexed by a statistically significant SNP, rs9296068, located near *HLA-DOA*. A three-SNP haplotype containing rs9296068 was significantly associated with childhood BCP-ALL.

We performed a 3-SNP sliding window haplotype analysis that included 395 genotyped SNPs across each of the 3 candidate regions (regions A, B, and C) identified by the single SNP analysis. After adjusting for multiple comparisons, a statistically significant association was found for the rs1237485-rs3118361-rs2032502 haplotype (nominal global p=3.2x10^-4^; corrected p=0.046) in region A (Table 3 and Figure 3A). Specifically, haplotype G-A-G was associated with an increased risk of BCP-ALL compared to other haplotypes combined (OR=2.18, 95% CI=1.41-3.38) (Table 3). In multivariable analysis adjusting for the nearby region A SNP, rs7747023 (described above in the individual SNP analysis), evidence of association for this haplotype remained strong (nominal global p=2.5x10^-3^), while the effect for rs7747023 appeared to be attenuated. When rs7747023 was included in the haplotype, the global test for the 4-SNP haplotype (rs1237485-rs3118361-rs2032502-rs7747023) yielded stronger evidence of an association (nominal global p=2.7x10^-5^). Stratified analyses by race/ethnicity showed similar results in non-Hispanic white and Hispanic children.

**Table 3:** Results for two associated regions based on a 3-SNP haplotype sliding window analysis of BCP-ALL cases and controls.

	Frequency		Compare to reference haplotype		Compared to all other haplotypes			
Haplotype	Cases (%)	Controls (%)	OR	95% CI^a^	OR	95% CI^a^	p-value	p-value_*(FWE)*_^b^
**Region A**								
rs1237485-rs3118361-rs2032502^c^								
A-A-G	0.06	0.06	1.03	(0.72-1.48)	1.16	(0.82-1.62)	0.406	
A-G-A	0.10	0.13	0.84	(0.65-1.08)	0.74	(0.58-0.94)	0.015	
A-G-G	0.37	0.33	1.22	(1.02-1.45)	1.14	(0.97-1.34)	0.109	
G-A-G	0.06	0.03	2.44	(1.54-3.88)	2.18	(1.41-3.38)	2.9 x10^-4^	
G-G-G	0.41	0.45	*1.00*	*Ref*	0.89	(0.76-1.04)	0.136	
						Global p-value^d^	3.2x10^-4^	0.046
**Region C**								
rs423639-rs7754316-rs9296068^c^								
A-A-A	0.07	0.08	0.82	(0.61-1.11)	0.93	(0.69-1.23)	0.576	
G-A-A	0.27	0.32	0.77	(0.63-0.93)	0.80	(0.67-0.95)	9.1x10^-3^	
G-A-C	0.39	0.34	*1.00*	*Ref*	1.25	(1.07-1.46)	6.0x10^-3^	
G-G-A	0.24	0.25	0.82	(0.67-1.00)	0.91	(0.76-1.10)	0.331	
G-G-C	0.04	0.02	1.98	(1.08-3.63)	2.47	(1.38-4.43)	1.7x10^-3^	
						Global p-value^d^	9.2 x10^-5^	0.014

Abbreviations: CI, confidence interval; FWE, family-wise type I error; OR, odds ratio; SNP, single nucleotide polymorphism

^a^ ORs and 95% CI for each haplotype were derived using logistic regression modeling haplotype probabilities and adjusting for child’s age, sex, and race/ethnicity (non-Hispanic white versus Hispanic).

^b^ Adjustment for multiple testing was performed with 10,000 permutations of case-control status on 393 haplotype windows and using FWE rate of 0.05. An adjusted p-value of less than 0.05 was considered statistically significant.

^c^ The associated region A haplotype is located in the extended class I region and the SNPs are at chromosomal positions 29,002,323 (rs1237485), 29,006,266 (rs3118361), and 29,009,544 (rs2032502). The associated region C haplotype is located in the class II region and the SNPs are chromosomal positions 33,095,752 (rs423639), 33,095,976 (rs7754316), and 33,096,673 (rs9296068).

^d^ Global p-values were derived based on a likelihood ratio test of association based on the null hypothesis of no effect of any haplotype at that position.

Finally, haplotype rs423639-rs7754316-rs9296068 (Table 3 and Figure 3B) of region C was found to be statistically significant (nominal global p=9.2x10^-5^; corrected p=0.014), a locus also identified through the individual SNP analysis of rs9296068. Previously, we reported an association between childhood ALL risk and the *DP1* supertype of *HLA-DPB1* (comprised of *HLA-DPB1* alleles *01:01*, *05:01*, *50: 01*) [12], a class II gene located about 55 kb from the associated rs9296068 SNP. The two loci showed weak correlation (*r* ^2^<0.1) and the analysis adjusting for carriers of the *DP1* supertype indicated an independent effect for rs9296068 (OR=1.37, 95% CI=1.16-1.63, p=3.0x10^-4^).

## Discussion

In this study, we conducted a SNP-association analysis of childhood BCP-ALL compared with controls across a 4 Mb stretch of the xMHC in an attempt to pinpoint regions of potential involvement in susceptibility. The xMHC is a potentially strong candidate region for a role in genetic susceptibility to childhood ALL, a disease whose causation has been attributed to an inappropriate immune response to post-natal infection [2,24,25]. Using a validated panel of greater than 1,100 SNPs designed to capture the genetic diversity of this complex genomic region, we identified two loci associated with childhood BCP-ALL risk. After correction for multiple testing, we found a statistically significant increased risk associated with the minor allele of rs9296068 in proximity to the *HLA-DOA* gene. A second independently associated locus, represented by haplotypes comprised of SNPs rs1237485, rs3118361, and rs2032502, was identified using a haplotype sliding window analysis and is located in the extended class I region in proximity to the *TRIM2*7 gene.

The rs9296068 SNP is located in the 5’ untranslated region of the *HLA-DOA* gene about 11.3 kb from the first exon and resides within a region that is predicted to have promoter function [26,27]. *HLA-DOA* encodes the alpha subunit of the HLA-DO heterodimer and is selectively expressed in B-cells and thymic medullary epithelium [28]. HLA-DO interacts with HLA-DM to regulate peptide loading onto MHC class II molecules in a pH-dependent manner. While HLA-DM facilitates peptide binding by catalyzing the exchange between low and high affinity peptides, HLA-DO impedes this function by reducing class II-mediated presentation in general, and has the ability to skew the presented antigenic peptide repertoire in B cells [29]. Thus, a balanced expression between HLA-DM and DO is critical in controlling antigen presentation in B-cells.

Recently, HLA-DOA has been implicated in other disease association studies such as type 1 diabetes and chronic lymphocytic leukemia survival [30,31], and interestingly, the same rs9296068 SNP was reported in a multistage MHC association mapping study of pediatric liver transplant rejection [26]. Functional validation showed a nearly 3-fold higher intra-graft B-lymphocyte content in rejecting liver grafts among carriers of the risk allele compared to non-carrier rejecters. This SNP was also associated in a recent GWAS of rheumatoid arthritis, but its independence from the known *HLA-DRB1* effect was not described [32]. Further biological relevance of this SNP locus is supported by publicly available expression quantitative trait loci data, with one source indicating an rs9296068 allelic- dependent association with *HLA-DOA* gene expression in lymphoblastoid cell lines [26,33], and another showing associations with *HLA-DPB1* gene expression in purified B-cells and monocytes [34].

Previous examinations of the xMHC in childhood ALL have mostly been candidate gene studies that focused on the classical HLA genes (i.e. *HLA*-*A*, -*B*, and -*C*, and *HLA-DP*, -*DQ*, and -*DR*). The most consistent evidence of an association has been for HLA class II loci, including *HLA-DPB1* [10,12] and *HLA-DR* [8,35], genes relatively close in proximity to rs9296068 and *HLA-DOA*. However, due to the lack of genetic characterization of the surrounding regions in these studies, it could not be unambiguously determined whether those associations indicated a causal link with the HLA gene or whether the associations were an effect of LD with an adjacent causal locus. The availability of directly genotyped *HLA-DPB1* data allowed us to confirm that the rs9296068 association of the class II region is independent of the *HLA-DPB1* allelic associations previously reported in the CCLS and elsewhere [10,12]. We were unable to confirm this for *HLA-DQ* and *HLA-DR*, but the presence of major recombination activity and the weak correlation between rs9296068 and SNPs immediately upstream make it unlikely that the association originates from the DQ/DR loci [36].

Authors of a recent report using data extracted from a prior GWAS analysis concluded no substantive support for a major role of MHC genetic variation on childhood BCP-ALL risk [13]. Among the results, the strongest single SNP association was observed for rs3135034 which exhibited a nominal p-value of 0.0017, but was not statistically significant after correction for multiple testing. SNP rs3135034 is located about 20 kb downstream of *HLA-DOA*, and only 37 kb from rs9296068 in an intergenic region also in proximity to the bromodomain containing 2 (*BRD2*) gene. In our data, rs3135034 is weakly correlated (r^2^<0.1) with rs9296068 in both non-Hispanic whites and Hispanics and showed no association with childhood BCP-ALL. However, rs9296068 and rs3135034 flank a well-characterized strong meiotic recombination hotspot, *DNA3* [37], and it has been noted that etiological variants within a recombination hot spot may be impossible to identify using standard association strategies [38]. This suggests that the identification of these two SNPs in close physical proximity in two independent studies of MHC association with childhood ALL could indicate a true association with *HLA-DOA*, partially masked by *DNA3*.

Using a haplotype sliding window analysis, we identified a second independently associated locus which is localized to the extended class I region and is represented by a haplotype comprised of SNPs rs1237485, rs3118361, and rs2032502. This haplotype maps to the 5’ untranslated region of the *TRIM27* gene about 2.5 kb from the coding region and is greater than 1Mb from the nearest classical HLA locus (*HLA-A*). TRIM27 (also known as Ret finger protein, RFP) belongs to an expanding family of proteins that are characterized by a tripartite motif comprising Really Interesting New Gene (RING) and B-box zinc-binding domains, and a variable coiled-coil region [39]. They participate in a variety of critical biological processes including cell growth, tumor suppression, DNA damage signaling, senescence, apoptosis, stem cell differentiation, and immune response to infections. Recent evidence demonstrated that TRIM27 (and other TRIM proteins) may contribute to a repertoire of pathways through its function as both a small ubiquitin-like modifier (SUMO) protein and ubiquitin E3 ligase important in post-translational modification [40,41]. The p53 tumor suppressor and its principal antagonist murine double minute 2 (Mdm2) oncogene are among the several substrates of TRIM27 SUMO and ubiquitin E3 ligase activity. With respect to immune regulation, TRIM27 is thought to down-regulate the immune response at multiple levels, including inhibition of toll-like receptor activation of nuclear factor-kappaB (NF-κβ) and interferon regulatory factor 3 (IRF3) [42], and the ability to negatively regulate CD4+ T cells through inhibitory effects on KCa3.1 (calcium-activated potassium channel) protein activity [43,44].

We did not impute genotypes for additional xMHC SNP loci or classical HLA alleles because certain features of the current study made it suboptimal for implementation of imputation including, 1) uncertainty in the use of currently available reference panels for imputation in a recently admixed population [45,46], 2) a focus on the xMHC, a region of complex LD that shows varying degrees of heterogeneity even across sub-strata of individuals of European descent [47,48], and 3) a sample size comparably large for a study of a rare disease, but not statistically robust to a multiple comparisons burden that would be elevated by close to 10-fold with the additional loci. Thus, it is possible that associations were missed due to limited SNP coverage in certain regions.

The associations reported from these analyses were not identified in the previous GWAS [49,50,51,52,53]. Notably, our study was performed on a sample size comparable to that of the previous GWAS of childhood ALL, but with a substantially smaller multiple comparisons burden on statistical power due to its focus only on the xMHC. However, we acknowledge that statistical power may have been affected by combining non-Hispanic white and Hispanic children for the analysis. The success of the association mapping approach is highly dependent on the degree of LD between the genotyped SNP and the causal locus. A loss in precision would be expected if this LD between the SNP and causal locus differed across populations included in the analysis [54]. While described as a limitation, an advantage of our approach is that the detectable associations would likely only be those that showed a relationship in both race/ethnicity populations, which may add to the confidence in results. Accordingly, the associations reported in the current analysis showed consistency between non-Hispanic white and Hispanic children in stratified analyses.

Certain characteristics of the association mapping approach, namely the dependence on SNP coverage and the effect of multiple comparisons on statistical power, may have contributed to inconsistencies between results of the current study and associations reported in previous studies based on the candidate gene approach. As reviewed previously [55], the two approaches should be viewed as complementary strategies for identification of disease associated loci as they both have their respective strengths and weaknesses depending on the study being conducted. A review of the literature identified six xMHC childhood ALL candidate gene studies of non-classical HLA loci, and statistically significant associations have been reported for SNPs of the *HFE*, *HSPA1B* and *BAT3* genes [56,57,58]. None of the SNPs specifically examined in these studies were genotyped as part of the current mapping panel which precluded our ability to directly evaluate these previous associations. Indirect assessment of previously associated *BAT3* SNPs was possible through identification of proxy SNPs (*r* ^2^>0.8 in HapMap CEU) using publicly available resources [59], but evidence of an association was not observed in our study.

Any substantial effect of population stratification is likely to be minimal in the NCCLS due to the careful and detailed account of race and ethnicity obtained from the subjects and statistical adjustment. As described earlier, this is further supported by our previous report showing estimates of genetic ancestry (percent of European, Amerindian, and African ancestry) to be similar between cases and matched controls [16]. However, the effects of any potential difference in localized genetic ancestry within the MHC between cases and controls cannot be ruled out.

In this comprehensive examination of genetic variation across the xMHC, we provide evidence localizing potential disease susceptibility loci for childhood BCP-ALL to two regions, the extended class I near *TRIM27* and class II near *HLA-DOA*. Confirmation of these findings in future studies through fine-mapping and replication in other populations is warranted.

## Supporting Information

Figure S1 **Quantile-quantile plot of the expected versus observed -log_10_ (p-value) distribution in the analysis of 1,145 xMHC SNPs and childhood BCP-ALL risk.**
Association results were derived by logistic regression assuming a log-additive genetic model and adjusting for child’s age, sex, and race/ethnicity. The red line represents the plot where the observed distribution of the -log_10_ (p-value) is same as the expected distribution given the number of SNPs tested.
(TIF)

Figure S2 **Linkage disequilibrium (LD) plot of the twenty SNPs associated with BCP-ALL with a p-value of less than 0.01.**
The values displayed in the plot are correlation coefficients (*r^2^*) and the intensity of shading corresponds to the *D’* measure for each marker pair. The plot and LD measures were generated separately among non-Hispanic white control children (A) and Hispanic control children (B) using Haploview (http://www.broad.mit.edu/mpg/haploview).
(TIFF)

Figure S3 **Stratified analysis of childhood BCP-ALL and the potentially associated SNPs presented in Table 1 by race/ethnicity, sex, and age group, and subgroup analyses by major subtypes.**
Odds ratios (ORs, represented by boxes with the area of each box inversely proportional to the variance of the estimate) and 95% confidence intervals (CIs, error bars) were derived using logistic regression adjusting for child’s age, sex, and race/ethnicity depending on the stratification variable. The dashed vertical line represents the OR of the SNP in the analysis of BCP-ALL among all subjects and the width of the diamond is the corresponding 95% CI. *P*_homogeneity_ was on the basis of the Cochran’s Q test statistic. Abbreviations: Ca, number of case; cALL, common acute lymphoblastic leukemia; Co, number of controls.
(PDF)

Table S1 **Evaluation of the independent effects in the multivariable analysis of 20 xMHC SNPs potentially associated with childhood BCP-ALL (p-value < 0.01 in the singles SNP analysis).**
(PDF)
