# Supplementary figures and images for: SNP Association Mapping across the Extended Major Histocompatibility Complex and Risk of B-Cell Precursor Acute Lymphoblastic Leukemia in Children
Source: PLoS One. 2013 Aug 22;8(8):e72557. doi: 10.1371/journal.pone.0072557 (PMC3749982; doi:10.1371/journal.pone.0072557)

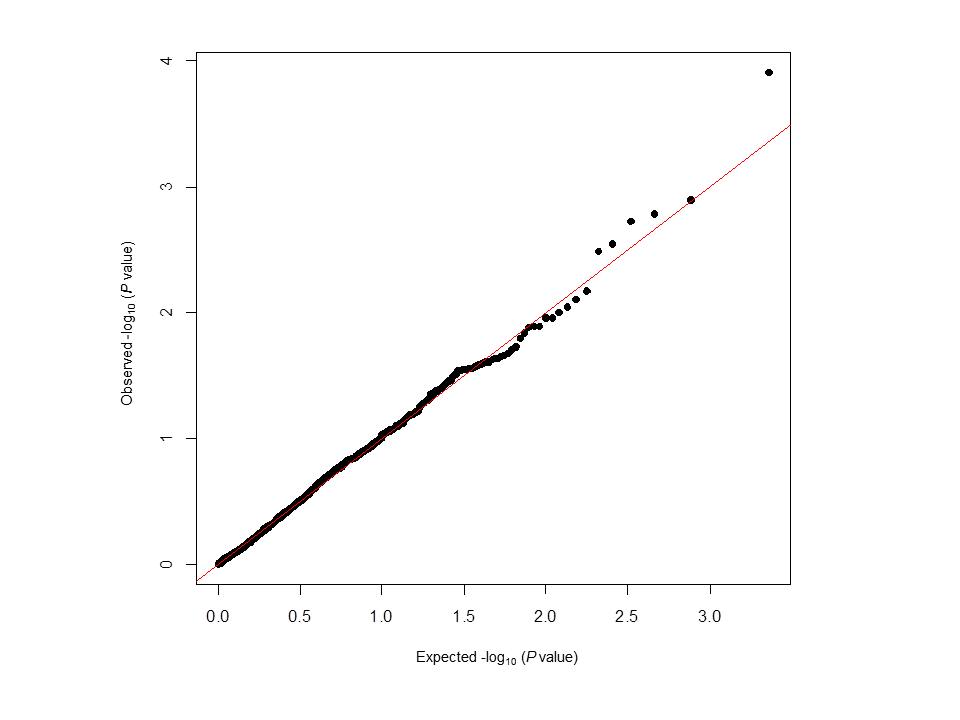

Supplement: Figure S1 — Quantile-quantile plot of the expected versus observed -log10 (p-value) distribution in the analysis of 1,145 xMHC SNPs and childhood BCP-ALL risk. Association results were derived by logistic regression assuming a log-additive genetic model and adjusting for child’s age, sex, and race/ethnicity. The red line represents the plot where the observed distribution of the -log10 (p-value) is same as the expected distribution given the number of SNPs tested. (TIF) [file pone.0072557.s001.tif]

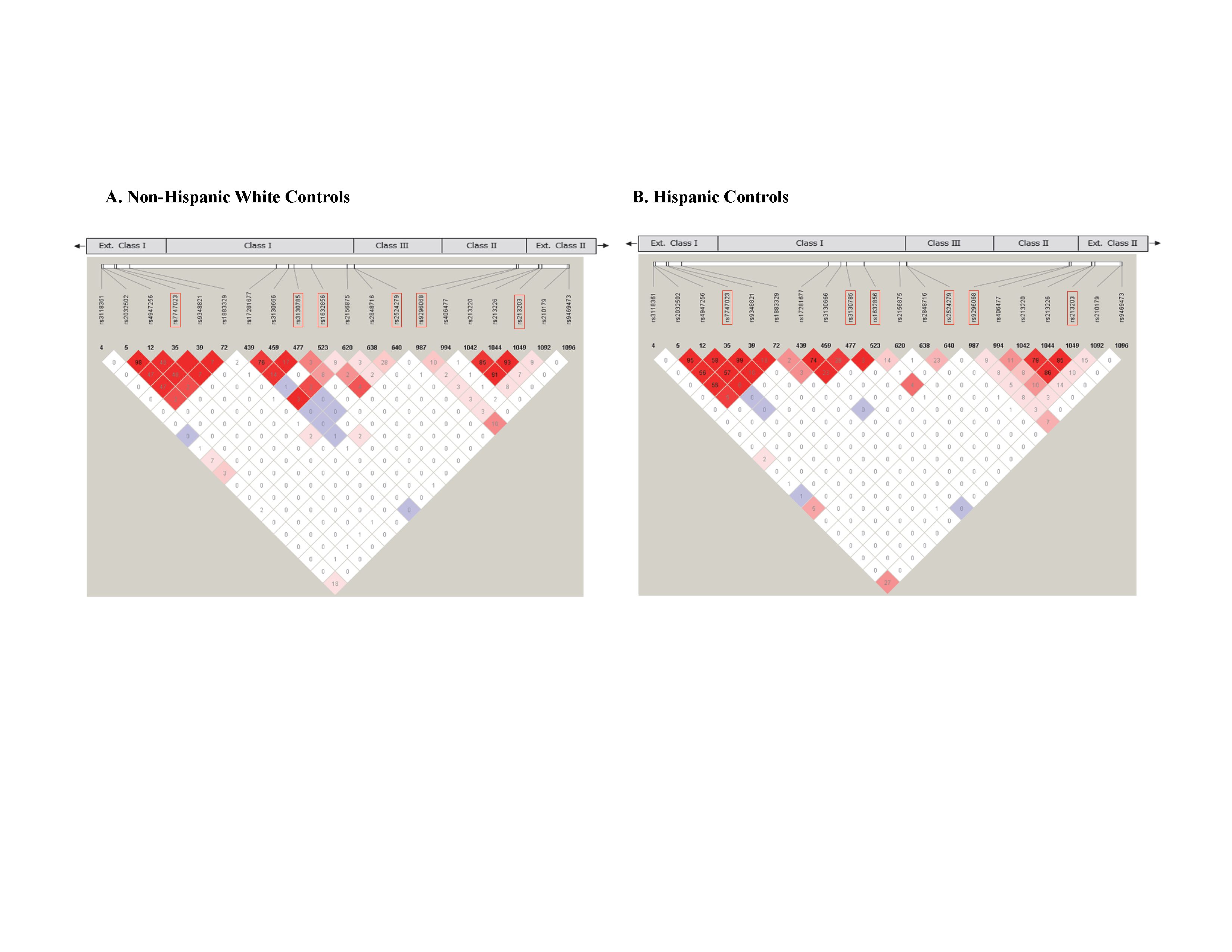

Supplement: Figure S2 — Linkage disequilibrium (LD) plot of the twenty SNPs associated with BCP-ALL with a p-value of less than 0.01. The values displayed in the plot are correlation coefficients (r2) and the intensity of shading corresponds to the D’ measure for each marker pair. The plot and LD measures were generated separately among non-Hispanic white control children (A) and Hispanic control children (B) using Haploview (http://www.broad.mit.edu/mpg/haploview). (TIFF) [file pone.0072557.s002.tiff]
