# Supplementary material for: SNP Association Mapping across the Extended Major Histocompatibility Complex and Risk of B-Cell Precursor Acute Lymphoblastic Leukemia in Children
Source: PLoS One. 2013 Aug 22;8(8):e72557. doi: 10.1371/journal.pone.0072557 (PMC3749982; doi:10.1371/journal.pone.0072557)

Extended Class I
rs7747023 (A>G)

	Ca	Co	OR	95%CI
**B-cell ALL: log-additive**	567	892	0.73	0.60-0.89
**Genetic Models**				
G/A (vs A/A)	145	285	0.70	0.56-0.87
G/G (vs A/A)	19	42	0.60	0.35-1.03
Dominant	164	327	0.69	0.55-0.87
Recessive	19	42	0.67	0.39-1.15
**By Race/Ethnicity (P homogeneity=0.106)**				
Non-Hispanic white	241	426	0.59	0.42-0.83
Hispanic	326	466	0.83	0.65-1.06
**By Sex (P homogeneity=0.735)**				
Male	298	495	0.71	0.54-0.93
Female	269	397	0.76	0.57-1.01
**By Age (P homogeneity=0.547)**				
0 - 5 years	391	571	0.75	0.60-0.94
6 - 14 years	176	321	0.66	0.47-0.93
**Major B-cell ALL subtypes**				
cALL (CD10+/CD19+, 2-5 yrs)	309	892	0.77	0.60-0.99
Non-cALL	258	892	0.71	0.54-0.93
Hyperdiploid	178	892	0.70	0.52-0.94
TEL-AML1	96	892	0.82	0.56-1.20
Normal karyotype	58	892	0.65	0.39-1.08

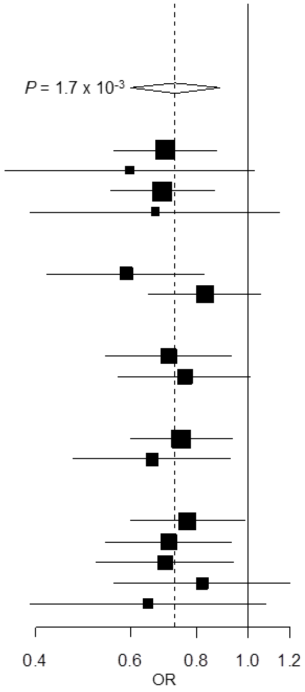

MHC Class I
rs3130785 (G>A)

	Ca	Co	OR	95%CI
**B-cell ALL: log-additive**	567	892	1.45	1.16-1.81
**Genetic Models**				
A/G (vs G/G)	147	181	1.46	1.13-1.89
A/A (vs G/G)	11	10	2.06	0.86-4.93
Dominant	158	191	1.49	1.16-1.91
Recessive	11	10	1.87	0.78-4.48
**By Race/Ethnicity (P homogeneity=0.194)**				
Non-Hispanic white	241	426	1.27	0.93-1.73
Hispanic	326	466	1.72	1.23-2.41
**By Sex (P homogeneity=0.596)**				
Male	298	495	1.38	1.01-1.89
Female	269	397	1.56	1.12-2.17
**By Age (P homogeneity=0.608)**				
0 - 5 years	391	571	1.39	1.04-1.86
6 - 14 years	176	321	1.57	1.09-2.26
**Major B-cell ALL subtypes**				
cALL (CD10+/CD19+, 2-5 yrs)	309	892	1.46	1.09-1.96
Non-cALL	258	892	1.40	1.05-1.87
Hyperdiploid	178	892	1.46	1.05-2.03
TEL-AML1	96	892	1.47	0.94-2.30
Normal karyotype	58	892	1.80	1.07-3.03

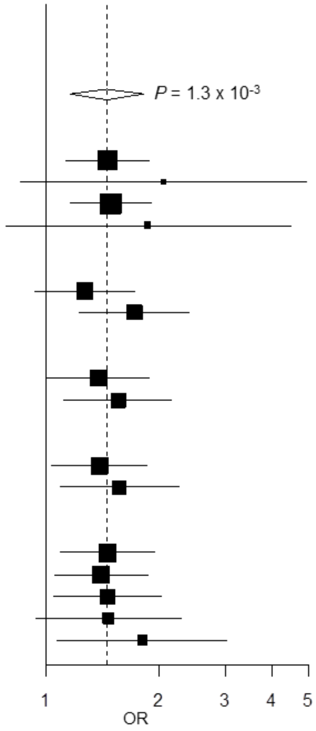

MHC Class I<br>rs1632856 (G>A)	Ca	Co	OR	95%CI
**B-cell ALL: log-additive**	567	892	0.80	0.68-0.94
**Genetic Models**				
A/G (vs G/G)	213	363	0.82	0.66-1.02
A/A (vs G/G)	35	80	0.62	0.40-0.96
Dominant	248	443	0.78	0.63-0.97
Recessive	35	80	0.67	0.44-1.02
**By Race/Ethnicity (P homogeneity=0.611)**				
Non-Hispanic white	241	426	0.84	0.66-1.07
Hispanic	326	466	0.77	0.61-0.97
**By Sex (P homogeneity=0.349)**				
Male	298	495	0.74	0.58-0.94
Female	269	397	0.87	0.69-1.10
**By Age (P homogeneity=0.947)**				
0 - 5 years	391	571	0.80	0.65-0.98
6 - 14 years	176	321	0.79	0.58-1.08
**Major B-cell ALL subtypes**				
cALL (CD10+/CD19+, 2-5 yrs)	309	892	0.82	0.66-1.02
Non-cALL	258	892	0.77	0.61-0.97
Hyperdiploid	178	892	0.74	0.57-0.96
TEL-AML1	96	892	0.75	0.53-1.06
Normal karyotype	58	892	0.66	0.42-1.04

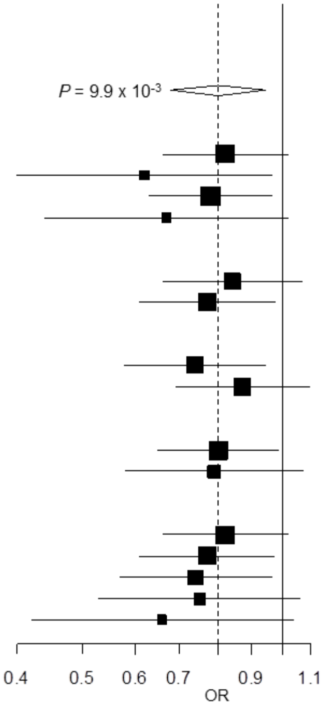

MHC Class I<br>rs2524279 (A>G)	Ca	Co	OR	95%CI
**B-cell ALL: log-additive**	565	888	0.73	0.58-0.92
**Genetic Models**				
G/A (vs A/A)	120	227	0.76	0.59-0.98
G/G (vs A/A)	4	16	0.36	0.12-1.08
Dominant	124	243	0.74	0.57-0.96
Recessive	4	16	0.34	0.13-0.89
**By Race/Ethnicity (P homogeneity=0.567)**				
Non-Hispanic white	240	424	0.67	0.46-0.98
Hispanic	325	464	0.77	0.57-1.04
**By Sex (P homogeneity=0.686)**				
Male	296	493	0.70	0.51-0.96
Female	269	395	0.77	0.55-1.08
**By Age (P homogeneity=0.699)**				
0 - 5 years	389	569	0.70	0.53-0.92
6 - 14 years	176	319	0.77	0.52-1.14
**Major B-cell ALL subtypes**				
cALL (CD10+/CD19+, 2-5 yrs)	308	888	0.77	0.57-1.04
Non-cALL	257	888	0.68	0.50-0.92
Hyperdiploid	178	888	0.66	0.45-0.97
TEL-AML1	95	888	0.58	0.34-0.99
Normal karyotype	58	888	0.99	0.58-1.69

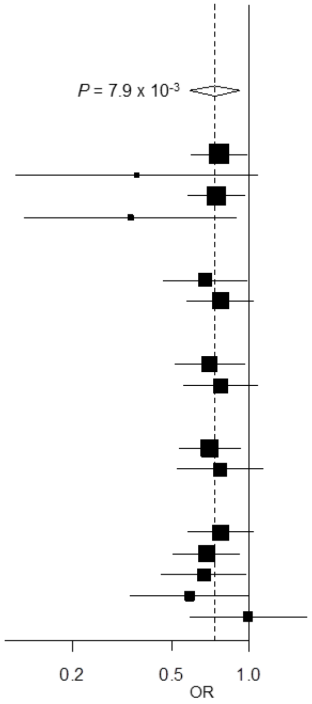

Extended Class II rs213203 (C>A)	Ca	Co	OR	95%CI
**B-cell ALL: A/C (vs C/C or A/A)**	247	471	0.68	0.55-0.84
**Genetic Models**				
A/C (vs C/C)	247	471	0.62	0.48-0.80
A/A (vs C/C)	146	210	0.83	0.62-1.11
Log-additive	567	892	0.90	0.78-1.04
Dominant	393	681	0.68	0.54-0.86
Recessive	146	210	1.12	0.88-1.43
**By Race/Ethnicity (P homogeneity=1.000)**				
Non-Hispanic white	241	426	0.68	0.49-0.94
Hispanic	326	466	0.68	0.51-0.91
**By Sex (P homogeneity=0.789)**				
Male	298	495	0.66	0.49-0.89
Female	269	397	0.70	0.51-0.96
**By Age (P homogeneity=0.655)**				
0 - 5 years	391	571	0.65	0.50-0.84
6 - 14 years	176	321	0.72	0.50-1.04
**Major B-cell ALL subtypes**				
cALL (CD10+/CD19+, 2-5 yrs)	309	892	0.69	0.53-0.90
Non-cALL	258	892	0.64	0.48-0.85
Hyperdiploid	178	892	0.67	0.48-0.94
TEL-AML1	96	892	0.76	0.50-1.16
Normal karyotype	58	892	0.40	0.22-0.73

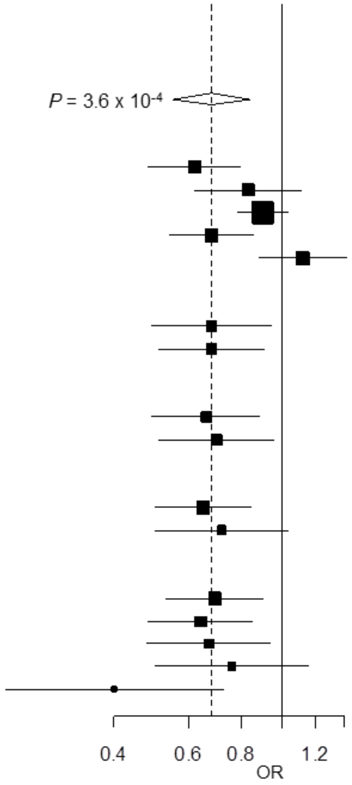

Supplement: Figure S3 — Stratified analysis of childhood BCP-ALL and the potentially associated SNPs presented in Table 1 by race/ethnicity, sex, and age group, and subgroup analyses by major subtypes. Odds ratios (ORs, represented by boxes with the area of each box inversely proportional to the variance of the estimate) and 95% confidence intervals (CIs, error bars) were derived using logistic regression adjusting for child’s age, sex, and race/ethnicity depending on the stratification variable. The dashed vertical line represents the OR of the SNP in the analysis of BCP-ALL among all subjects and the width of the diamond is the corresponding 95% CI. P homogeneity was on the basis of the Cochran’s Q test statistic. Abbreviations: Ca, number of case; cALL, common acute lymphoblastic leukemia; Co, number of controls. (PDF) [file pone.0072557.s003.pdf]
