# Supplementary material for: SNP Association Mapping across the Extended Major Histocompatibility Complex and Risk of B-Cell Precursor Acute Lymphoblastic Leukemia in Children
Source: PLoS One. 2013 Aug 22;8(8):e72557. doi: 10.1371/journal.pone.0072557 (PMC3749982; doi:10.1371/journal.pone.0072557)
Supplement: Table S1 — Evaluation of the independent effects in the multivariable analysis of 20 xMHC SNPs potentially associated with childhood BCP-ALL (p-value < 0.01 in the singles SNP analysis). (PDF) [file pone.0072557.s004.pdf]

Table S1. Evaluation of the independent effects in the multivariable analysis of 20 xMHC SNPs potentially associated with childhood BCP-ALL (p-value < 0.01 in the singles SNP analysis).

				Model							
				SNP + covariates		SNP + rs7747023 + covariates		SNP + rs3130785 + covariates		SNP + rs9296068 + covariates	
SNP	Position	Minor allele	Test[a]	OR (95% CI)[b]	p-value	OR (95% CI)[b]	p-value	OR (95% CI)[b]	p-value	OR (95% CI)[b]	p-value
**Region A**											
rs3118361	29006266	A	dom	1.45 (1.11-1.89)	$6.4x10^{-3}$	1.42 (1.09-1.85)	0.010	1.50 (1.14-1.95)	$3.2x10^{-3}$	1.34 (1.03-1.76)	0.032
rs2032502	29009544	A	dom	0.70 (0.53-0.91)	$7.5x10^{-3}$	0.88 (0.61-1.28)	0.513	0.70 (0.53-0.91)	$8.1x10^{-3}$	0.70 (0.53-0.91)	$7.6x10^{-3}$
rs4947256	29029919	A	add	0.72 (0.57-0.91)	$6.7x10^{-3}$	0.90 (0.63-1.27)	0.532	0.72 (0.57-0.92)	$8.1x10^{-3}$	0.72 (0.56-0.91)	$6.4x10^{-3}$
rs7747023[c]	29133659	G	add	0.73 (0.60-0.89)	$1.7x10^{-3}$	NA	NA	0.73 (0.60-0.89)	$1.6x10^{-3}$	0.73 (0.60-0.88)	$1.4x10^{-3}$
rs9348821	29155182	G	add	0.74 (0.61-0.89)	$1.9x10^{-3}$	1.03 (0.14-7.79)	0.977	0.73 (0.60-0.89)	$1.7x10^{-3}$	0.73 (0.60-0.89)	$1.6x10^{-3}$
rs1883329	29285081	A	dom	1.34 (1.09-1.66)	$6.7x10^{-3}$	1.24 (0.99-1.54)	0.060	1.34 (1.08-1.65)	$7.7x10^{-3}$	1.33 (1.08-1.65)	$8.6x10^{-3}$
**Region B**											
rs17281677	30730438	A	add	1.76 (1.15-2.70)	$9.0x10^{-3}$	1.71 (1.12-2.63)	0.013	1.39 (0.87-2.22)	0.173	1.82 (1.19-2.80)	$6.1x10^{-3}$
rs3130666	30848139	A	add	1.80 (1.22-2.66)	$3.3x10^{-3}$	1.78 (1.20-2.64)	$4.0x10^{-3}$	1.46 (0.94-2.26)	0.095	1.85 (1.24-2.74)	$2.4x10^{-3}$
rs3130785[c]	30904717	A	add	1.45 (1.16-1.82)	$1.3x10^{-3}$	1.46 (1.16-1.83)	$1.2x10^{-3}$	NA	NA	1.46 (1.16-1.83)	$1.2x10^{-3}$
rs1632856	31079715	A	add	0.80 (0.68-0.95)	$9.9x10^{-3}$	0.79 (0.67-0.94)	$7.9x10^{-3}$	0.83 (0.70-0.99)	0.038	0.78 (0.66-0.93)	$4.7x10^{-3}$
rs2156875	31425326	A	rec	0.70 (0.54-0.91)	$7.6x10^{-3}$	0.69 (0.53-0.90)	$5.8x10^{-3}$	0.75 (0.57-0.97)	0.031	0.67 (0.52-0.88)	$3.6x10^{-3}$
rs2848716	31495946	G	rec	0.37 (0.18-0.76)	$6.2x10^{-3}$	0.37 (0.18-0.75)	$5.7x10^{-3}$	0.40 (0.20-0.81)	0.011	0.37 (0.18-0.76)	$6.2x10^{-3}$
rs2524279	31500885	G	add	0.73 (0.58-0.92)	$8.0x10^{-3}$	0.73 (0.58-0.92)	$7.4x10^{-3}$	0.74 (0.58-0.93)	0.010	0.73 (0.58-0.92)	$8.3x10^{-3}$
**Region C**											
rs9296068[c]	33096673	C	add	1.37 (1.17-1.61)	$1.2x10^{-4}$	1.38 (1.17-1.62)	$1.0x10^{-4}$	1.38 (1.17-1.62)	$1.2x10^{-4}$	NA	NA
rs406477	33113622	G	rec	2.03 (1.21-3.42)	$7.7x10^{-3}$	2.10 (1.24-3.54)	$5.7x10^{-3}$	1.92 (1.14-3.24)	0.015	1.70 (1.00-2.87)	0.052
rs213220	33310618	G	dom	0.69 (0.54-0.88)	$2.4x10^{-3}$	0.69 (0.54-0.87)	$2.1x10^{-3}$	0.69 (0.54-0.88)	$2.6x10^{-3}$	0.76 (0.60-0.98)	0.032
rs213226	33317288	G	rec	1.39 (1.09-1.78)	$8.4x10^{-3}$	1.40 (1.09-1.79)	$8.3x10^{-3}$	1.39 (1.08-1.78)	$9.4x10^{-3}$	1.27 (0.99-1.64)	0.064
rs213203[d]	33346382	A	het	0.68 (0.55-0.84)	$3.6x10^{-4}$	0.69 (0.56-0.85)	$6.0x10^{-4}$	0.68 (0.55-0.84)	$4.0x10^{-4}$	0.68 (0.55-0.84)	$4.0x10^{-4}$
rs210179	33593214	A	rec	1.43 (1.11-1.85)	$6.3x10^{-3}$	1.45 (1.12-1.87)	$4.7x10^{-3}$	1.42 (1.10-1.83)	$7.8x10^{-3}$	1.35 (1.04-1.75)	0.022
rs9469473	33610865	G	add	1.42 (1.13-1.78)	$2.9x10^{-3}$	1.41 (1.12-1.78)	$3.0x10^{-3}$	1.44 (1.15-1.82)	$1.7x10^{-3}$	1.31 (1.04-1.65)	0.023

Abbreviations: CI, confidence interval; NA, not applicable; OR, odds ratio; SNP, single nucleotide polymorphism; xMHC, extended major histocompatibility complex

[a]The genetic model of inheritance assumed in the logistic regression model and corresponding to the presented results. Abbreviations: add, log-additive; dom, dominant; rec, recessive; het, heterozygotes versus homozygotes

[b]ORs and 95% CI for each SNP were derived using logistic regression assuming the indicated genetic model of inheritance and adjusting for child's age, sex, race/ethnicity (non-Hispanic white versus Hispanic), and additionally for either rs7747023, rs3130785, or rs9296068 as specified in the table.

[c]The 20 SNPs showing a p-value of less than 0.01 in the single SNP analysis appeared to spatially cluster within 1 of 3 regions of the xMHC. Each SNP was adjusted for rs7747023, rs3130785, and rs9296068, the SNP with strongest p-value within each region, to evaluate the independence of effects.

[d]Evaluation of the genetic model of inheritance indicated a significant deviation from the log-additive model with an effect associated with heterozygotes. ORs and 95% CI were estimated for heterozygous genotypes compared to homozygous genotypes.
